# Helpers' Self-Assessment Biases Before and after Helping Skills Training

**DOI:** 10.3389/fpsyg.2017.01377

**Published:** 2017-08-15

**Authors:** Marine Jaeken, Emmanuelle Zech, Céline Brison, Lesley L. Verhofstadt, Nady Van Broeck, Moïra Mikolajczak

**Affiliations:** ^1^Psychological Sciences Research Institute, Université Catholique de Louvain Louvain-la-Neuve, Belgium; ^2^Department of Experimental Clinical and Health Psychology, Ghent University Ghent, Belgium; ^3^Department of Clinical Psychology, KU Leuven Leuven, Belgium

**Keywords:** exploration helping skills, helping skills training, self-diminishment bias, self-enhancement bias, skilled-unaware pattern

## Abstract

Several studies have shown that therapists are generally biased concerning their performed helping skills, as compared to judges' ratings. As clients' ratings of therapists' performance are better predictors of psychotherapy effectiveness than judges' ratings, this study examined the validity and effectiveness of a helping skills training program at reducing novice helpers' self-enhancement biases concerning their helping skills, in comparison to their clients' ratings. Helping skills were assessed by three objective measures (a knowledge multiple choice test, a video test and a role play), as well as by a self- and peer-reported questionnaire. In addition, some performed helping skills' correlates (relationship quality, session quality, and helpers' therapeutic attitudes) were assessed both by helpers and their simulated helpees. Seventy-two sophomores in psychology participated to this study, 37 being assigned to a 12-h helping skills training program, and 35 to a control group. Helpers were expected to assess the aforementioned performed helping skills and correlates as being better than their helpees' assessments at pretest, thus revealing a self-enhancement bias. At posttest, we expected that trained helpers would objectively exhibit better helping skills than untrained helpers while beginning to underestimate their performance, thus indexing a self-diminishment bias. In contrast, we hypothesized that untrained helpers would continue to overestimate their performance. Our hypotheses were only partly confirmed but results reflected a skilled-unaware pattern among trainees. Trained helpers went either from a pretest overestimation to a posttest equivalence (performed helping skills and performed therapeutic attitudes), or from a pretest equivalence to a posttest underestimation (performed session quality and performed therapeutic relationship), as compared to helpees' ratings. Results showed that trained helpers improved on all helping skills objective measures and that helpees' perceptions of their performance had increased at posttest. In conclusion, helping skills training leads helpers not only to improve their helping skills but also to have more doubts about their skills, two variables associated with psychotherapy outcome.

## Introduction

Studies have found that some therapists lead to better client outcomes than others (Okiishi et al., 2003). This phenomenon, called the “therapist effect,” remains poorly understood but is at least partly explained by the therapist's influence on the therapy relationship (Anderson et al., 2009). By their way of communicating, therapists can either facilitate or undermine alliance and empathy, two core elements of the therapeutic relationship that substantially contribute to psychotherapy outcomes (Norcross and Wampold, 2011). For example, by reflecting the client's feelings and thoughts, the therapist demonstrates some empathy, which enhances both the therapeutic alliance (Moyers and Miller, 2013) and the psychotherapy outcomes if this empathy is perceived by the client (Elliott et al., 2011). Although grounded in the psychotherapy's field, this kind of supportive communication is not reserved to experienced psychotherapists (Tracey et al., 2014) and is also desirable for psychologists who are entitled to professionally help clients (Hatcher, 2015). Research has shown that interpersonal skills can be mastered by psychology students after a short training course (Hill et al., 2008; Kuntze et al., 2009). Since this study focuses on the training of undergraduates in psychology, the words “helpers” and “helpees” which refer to more basic helping situations will be used instead of “therapists” and “clients.”

Depending on the authors, professional helpers' supportive communication has been called facilitative interpersonal skills (Anderson et al., 2009), counseling communication skills (Kuntze et al., 2007), or helping skills (Hill et al., 2008). To best reflect their therapeutic aim, this article will use the term “helping skills,” viewed as therapists' exploratory interventions such as restatements and reflections of feelings (Hatcher, 2015). Helping skills are a complex construct that can be apprehended both at an objective (e.g., the skill is performed, observed and coded by a judge) and a subjective level (e.g., perceptions about the skill). According to Miller's pyramid (Miller, 1990), the objective assessment of a skill requires the evaluation of four levels of competency of increasing complexity: (1) theoretical knowledge about the skill (know), (2) knowledge of how to apply the skill knowledge in a concrete situation (know-how), (3) using the skill in a situation close to reality (show-how), and (4) using the skill in a real professional situation (do). However, the different levels are found to be poorly inter-correlated and lower levels do not necessarily predict upper levels (Crossley et al., 2002; ten Cate, 2006). Indeed, as mentioned by Brasseur et al. (2013), knowing something does not imply being able to apply it, and even if one is able to apply it, one may not necessarily do it when appropriate. Moreover, for a same skill, these four objective levels are poorly linked to subjective self- and peer-reports as well as to psychotherapy effectiveness (Hill et al., 2008).

Self-report measures of helping skills are used in most research but are problematic (Anderson et al., 2016). In general, in comparison to their observed skills, when people assess their skills, they tend to display a “self-enhancement bias” (Gosling et al., 1998) leading them to lack accuracy when judging their own competences and to overestimate themselves. More precisely, Burson et al. (2006) have shown that when people perform poorly as assessed objectively, they are more inclined to overestimate their performance (self-enhancement bias). On the contrary, those who perform better tend to underestimate their performance (self-diminishment bias), especially when performing a difficult task (Burson et al., 2006). This effect is called the skilled-unaware pattern (Kruger and Dunning, 1999). In fact, therapists seem to be inaccurate about their skills and subject to cognitive biases and positivity distortions (Macdonald and Mellor-Clark, 2015). Macdonald and Mellor-Clark (2015) reviewed several studies showing that therapists systematically overestimate their effectiveness and underestimated their clients' deterioration rate. Concerning helping skills specifically, three studies have shown that, in comparison to judges' evaluations, undergraduates tended to overestimate their skills before being trained but then to underestimate these after the training (Urbani et al., 2002; Little et al., 2005; Lepkowski et al., 2009).

Beyond the low validity aspect of therapists' self-reported helping skills, therapists' overestimations of themselves can prevent them from recognizing that their clients are experiencing difficulties and may begin to deteriorate or drop out (Walfish et al., 2012; Macdonald and Mellor-Clark, 2015). Nissen-Lie et al. (2013) have shown that therapists who had doubts about their ability to help their clients, what they called “professional self-doubt,” was positively associated with alliance and client outcomes. These results were replicated in 2015 and showed that self-overconfident therapists did not create a healthy therapeutic attitude (Nissen-Lie et al., 2015). These authors explain that therapists' uncertainty is a kind of wisdom and that their awareness of the helping complexity is favorable for their professional development. It seems therefore desirable for clients' outcomes that therapists view their skills as more restrictive than they are in reality.

Objective and self-reported measures of helping skills are poorly correlated, produce different results and suffer from several methodological limitations (Hill et al., 2008; Anderson et al., 2016). The literature on skills self-assessment (Gosling et al., 1998) and on helpers' biases concerning their helping skills (Urbani et al., 2002; Little et al., 2005; Lepkowski et al., 2009) compared self-ratings to observed behavior and considered the latter as the most reliable indicator (reflecting the “real skill”). The bias is conceived as the distance between self-assessments and objective assessments. However, we have shown in another study that judges' ratings of helpers' helping skills are not correlated with helpees' ratings (Jaeken et al., submitted). Since it is the clients' perception of therapists' empathy and helping skills that best predicts psychotherapy effectiveness (Elliott et al., 2011), and as the need for improving therapists' helping skills is in most cases justified by their link with clients' outcome, helpees' assessments will be considered as the rating reference in this study.

To our knowledge, little research has been conducted to examine inexperienced helpers' biases in the self-assessment of their interpersonal skills before and after a helping skills training (Urbani et al., 2002; Little et al., 2005; Lepkowski et al., 2009). Even less examine this kind of bias with reference to helpees' ratings. The aforementioned scientific literature lead us to think that novice helpers should overestimate their helping skills before having been trained (Gosling et al., 1998; Urbani et al., 2002; Little et al., 2005; Lepkowski et al., 2009). The same literature makes us expect that by becoming more helping skilled and therefore performing better during the difficult task of helping a simulated client in a session, helpers should demonstrate a self-diminishment bias after the training when assessing their helping skills (Urbani et al., 2002; Little et al., 2005; Burson et al., 2006; Lepkowski et al., 2009). By participating to the training, helpers will discover the complexity of helping and become less confident about their ability to effectively help their helpees, which amounts to enhancing their professional self-doubt (Nissen-Lie et al., 2013). It should be especially true for inexperienced novice helpers who experience considerable confusion and anxiety when learning new skills and theories (Stoltenberg and McNeill, 2010). Finally, since a way of reducing therapists' overconfidence is getting feedback about their behavior (Tracey et al., 2014; Macdonald and Mellor-Clark, 2015), a training including some feedback about helpers' helping skills, especially from their helpees, should also foster their professional self-doubt.

In order to compare helping skills self-ratings from helpers to ratings by their helpees and independent judges before and after helping skills training, we looked for a training course that: (1) had proved its effectiveness, (2) targeted undergraduates in psychology, (3) aimed to develop the exploration helping skills regardless of theoretical orientations in psychology, (4) included supervised role plays, (5) was short in duration, (6) was associated to published training material (at least a detailed book). Two training courses met these criteria: the cumulative micro training (CMT) (Kuntze et al., 2009) and the Hill model of helping skills training (Hill, 2009). The CMT program is a very structured way of enhancing exploration helping skills among psychology undergraduates (Kuntze et al., 2009). The program contains five sessions where each skill is learned separately in six steps and then integrated with the previous one. First, the targeted helping skill and its function are explained theoretically. Second, the use of the skill is illustrated by a “bad” and a “good” video example. Third, students have to use the skill during a short oral exercise. Fourth, students use the helping skill in a role-play with another student. Fifth, the student who played the helper receives feedback from both the student who played the helpee and the trainer. In the last step, students have to write down a feedback in order to get better during the following skill training session. Helping skills are thus learned separately, but they are gradually integrated. The Hill model of helping skills training (Hill, 2009) includes three components: (1) a skill is given as a lecture and then discussed in group (1 h per week), (2) students read and discuss articles about helping skills (1 h per week), and (3) each skill is practiced in small groups of students (2 h per week). Besides these 4 h of training per week, students have to conduct a 20-min helping session with another student playing the helpee. These role-plays take place at the beginning and during two thirds of the way through the course, respectively. The Hill training course lasts 4 h a week spread over 15 weeks (Hill, 2009).

Since both the CMT and Hill training programs met our criteria, we decided to combine them into a 12-h program (due to time constraint). As its very structured method appeared well suited to a short course, we kept the CMT components (skills, video examples, role plays). We added the theoretical content about the exploration stage from the Hill helping skills model because it had been evaluated as having the best coverage with regard to skills, culture, theory, cognition and affect, and relationship to therapeutic change (Hill et al., 2015). In order to remain within the time constraints, our helping skills course was limited to the *Exploration* level of the psychotherapeutic process (see Hill et al., 2008).

Based on previous research, the aim of this study was to examine the validity and effectiveness of a helping skills training program both at improving novice helpers' helping skills and at reducing their self-enhancement bias. Firstly, we wanted to test whether helpers improved their helping skills after the training, as assessed by different objective and subjective helping skills measures. We expected an improvement on all objective and subjective helping skills measures (hypothesis 1). Secondly, we examined the training effects on the potential self-enhancement bias of novice psychologists. As improved helping skills lead to enhanced performed session quality, better performed therapeutic relationship, and performed therapeutic attitudes during the role play (Hill et al., 2008), we also wanted to examine helpers' potential self-assessment bias on the correlates of these helping skills. Prior to training, we expected helpers to systematically evaluate their helping skills and their correlates better than helpees would rate them (self-enhancement bias) (hypothesis 2.1). At posttest, we predicted the opposite, with a significantly lower assessment of the variables by helpers than by their helpees (self-diminishment bias) (Hypothesis 2.2).

## Materials and methods

### Participants

Seventy-two second-year undergraduate psychology students participated in this study in exchange for course credit. The training group consisted of 37 students (31 females and 6 males; ranging in age from 18 to 34 years old, *M* = 20.24, *SD* = 2.69) and the control group of 35 students (28 females and 7 males; ranging in age from 18 to 25 years old, *M* = 19.80, *SD* = 1.64). Students were recruited by invitation to an information session where they received all the practical information (e.g., training duration and dates, assessments duration). An enrolment sheet containing 40 places for the helping skills training course was put up at the end of the information session. Registration was on a first come first served basis. Three students did not show up to the first training session. To facilitate interactions during training sessions, the remaining 37 students were randomly divided into two training subgroups of 18 and 19 respectively. Trained students rated their motivation to participate to the training at 7.51 (*SD* = 1.17) on a scale from 1 to 10. They were also asked to report their main motivation to participate: 81% (*N* = 31) wanted to learn helping skills and to add it on their CV, while 2.7% (N = 1) reported being motivated by course credits. At the end of the study, they received a 5-h credit that in fact rewarded their experimental participation for 14 h (assessments and training duration) as well as a certificate of training participation. Three months later, the control group was constituted. Second-year undergraduate psychology students who had not participated to the training experiment were recruited to participate to a new experiment. It proposed to offer them a feedback about their empathy and a 2-h credit in exchange of their participation to helping skills assessments (2 h). Students received no training but a copy of their client's evaluation about their empathy (ES; Burns et al., 1996) after the posttest measures. We also opened 40 places, but only 35 students completed both pre- and posttest measures. This two-step recruitment strategy was used to avoid a higher drop out of students in the control condition at posttest. In that condition indeed they could, in comparison to those in the training condition, be more disappointed not to receive the training by being randomized to a no training control group.

This study shared control group's recruitment and assessment procedures with another that is presented elsewhere (Brison et al., 2015). The Psychological Sciences Research Institute's ethical committee approved the study. This study was carried out in accordance with the recommendations of Psychological Sciences Research Institute's ethical committee with written informed consent from all subjects. All subjects gave written informed consent in accordance with the Declaration of Helsinki.

### The helping skills training course

Our training course aimed at improving the exploration helping skills of bachelor students in psychology with no experience or knowledge of helping sessions. We selected the seven basic helping skills mentioned by Kuntze et al. (2009) which are very general and fit all psychology orientations. Besides, although sometimes named differently, these are the most frequently-reported skills in the scientific literature (e.g., Spitzberg et al., 1990; Hill, 2009; Hargie, 2011). According to Lang and van der Molen (2009), basic helping skills are used at the beginning of the helping process to explore and clarify the helpee's problems.

As mentioned above, the training method is a short adaptation and combination of two effective programs: the Cumulative Microtraining method (CMT) developed by Lang and van der Molen (1992) and the helping skills training designed by Hill et al. (2008). In our course, each helping skill is taught in three steps. Firstly, the students receive theoretical information about the skill and its role in a helping session. Then, they apply the theory by completing short written exercises, for example transforming a closed question into an open one. During the second step, students are shown two video examples of the skill being used in a helping session. The first video illustrates poor use of the skill and the second good use. After each video example, students discuss what they liked or disliked about the helper's behavior with the trainer. Finally, the third step consists of practicing the skill in role plays. The students form groups of three and share out the roles of helper, helpee, and observer. In order to facilitate the helper's use of the targeted skill, the helpees receive a written description of a life difficulty to role-play for each helping skill. The role play lasts about 10 min and then role players and observers exchange their impressions in a debriefing session. Students then exchange roles so that they all play each role once. The trainer moves among the subgroups giving feedback and the exercise ends with a debriefing session with the whole group. A new helping skill is then learnt according to the same steps (theory—video—role plays), and this is finally integrated with those learned previously.

The helping skills training course lasted 12 h divided into four 3-h sessions given every week over 1 month. Each session targeted two helping skills learned in an ascending complexity order (1° minimal encouragements and asking questions, 2° paraphrasing and reflection of feeling, 3° concreteness and summarizing, and 4° situation clariflcation). The last session targeted situation clarification's skill and provided a summary of the three previous, ending with a debriefed longer role play were trainees could use all learned helping skills they found relevant. Trainers^1^ were two 5th year undergraduate psychology students (both 22 year-old females) who gave the helping skills course together in the two subgroups.

### Measures

**Figure 1:**
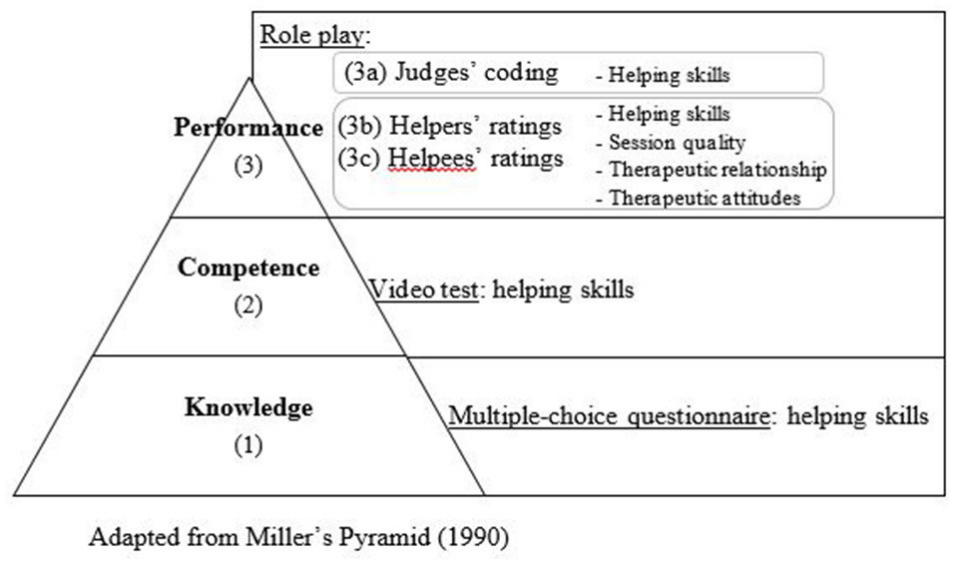
Measures.

As explained in detail below and summarized in Figure 1, helping skills were assessed using both objective and subjective measures. The helping skills' objective assessment was based on Miller's pyramid (Miller, 1990) with the methods proposed by Smit and van der Molen (1996): (1) theoretical knowledge was assessed by a multiple-choice questionnaire, (2) competence was assessed by a video test, and (3) performance during a role play was coded by independent judges (3a). Helpers' performance during the role play was also subjectively rated by themselves (3b) and their helpees (3c) at four levels: helping skills (*Helping Skills Measure-Exploration)*, therapeutic relationship *(Relationship Scale)*, session quality *(Session Evaluation Scale)*, and helper's therapeutic attitudes (empathy, warmth, genuineness) *(Empathy Scale)*.

All measures were completed before and after the course at a 6-week interval, with the same interval in the control group. Participants had collective assessment sessions in our laboratory where they answered the multiple-choice questionnaire and undertook the video test. Afterwards, they had individual appointments where they took part in a role-play with a simulated helpee (a confederate of the experimenter) and had to perform helping skills. Just after the role play, the helper and the helpee rated separately helper's performance at the four assessed levels.

#### The helping skills' knowledge test (multiple choice questionnaire)

In order to assess students' theoretical knowledge about the seven exploration helping skills (first level of Miller's pyramid, Miller, 1990), we used a multiple choice questionnaire based on Hill (2009). It contains 10 questions, each with 5 possible answers. An example is: “When a client remains very vague about his/her problem, the psychologist can: (1) reflect the client's feelings, (2) paraphrase what the client has said, (3) summarize what the client has said, (4) invite the client to give a concrete example of what he/she is talking about, and (5) all of the above. As in this example, all answers could be correct for some questions. Correct answers were summed, leading to a total out of ten, with higher scores indicating higher levels of helping skills' knowledge. The same version was used at pre- and posttest. Similar tests exist in English and have good efficiency, reliability, and validity (e.g., Smit and van der Molen, 1996; Hill, 2009).

#### The helping skills' competence assessment

To assess the competence level of acquisition of exploration helping skills, a French video test based on the Communication Skills Progress Test (CSPT, Kuntze et al., 2007) was used. This French version keeps the CSPT logic but contains other helpees, stories and situations (with the authors' agreement). The test exists in two versions (A and B) of seven video extracts (of about twenty seconds each), with the same level of difficulty. In each extract, a filmed helpee (played by a professional actor) expresses him/herself concerning a personal problem (of average importance, e.g., “I came today because I am exhausted—silence—I can't sleep anymore, and I lack energy all day long”) in front of the camera, giving the impression that he/she is talking to the audience. Students are asked to imagine themselves as the helper and to write down directly what they would say to the helpee if they were in a real interaction. They have 3 min after each extract to write down their answer on a sheet of paper. At pretest, a randomly selected half of the participants completed version A, while the other half completed version B. At posttest, the participants completed the version other than the one they had done for the pretest.

#### The competence coding procedure

The answers were coded by two independent judges^2^ (two female students of 26 and 22 years old in 4th year of psychology, trained to code the video test as part of their research internship). It was a blind coding as they did not know if it was pre- or posttest, nor if participants were in the control or training group. Each sentence was coded into one of the seven exploration helping skills. As in the Helping Skills System (HSS; Hill and O'Brien, 1999), to get the proportion of exploration helping skills used in the video test, we divided the exploration helping skills by the total number of skills used, higher scores indicating higher use of helping skills compared to the total of their utterances in the video test. As presented in Table 1 and similarly to Kuntze et al.'s (2009) findings, interrater reliabilities were satisfactory for the 7 skills. At pretest, students barely used Summarizing, such that an intra-class correlation coefficient could not be calculated.

**Table 1:** Intra-class correlation coefficients for helpers' helping skills used during the video test, as coded by independent judges.

	**Pretest**	**Posttest**
1. Minimal encouragement	0.88	0.99
2. Asking questions	0.95	0.93
3. Paraphrasing	0.95	0.95
4. Reflection of feeling	0.89	0.82
5. Concreteness	0.91	0.87
6. Summarizing	/	0.79
7. Situation clarification	0.83	0.92

### The helping skills' performance assessment

To assess performance level, we asked students to take part in a filmed role play (with preliminary consent) where they had to play the helper with a standardized helpee. The helpee was played by two experimenters (two 22-year old female students in 5th year of psychology working for their master's thesis, unknown by the students and trained to play standardized helpees and to administer the different measures). They were responsible for the evaluation sessions at pre- and posttest. They created two helpee's scenarios of equivalent difficulty (Version A: a teacher exhausted by her students, version B: an employee exhausted by a colleague). The two helpees' versions and experimenters were crossed at pre- and posttest: a randomly selected half of the participants interacted in role-play A at pretest while the other half interacted in role-play B, reversing this in the posttest. Students received as the only instruction “*play the helper for five minutes as if it were a real helping session with a real helpee coming to see you*.” The role play lasted 5 min at the most (a bell signaled time-up) but students could stop before if they did not know what to say and felt uncomfortable.

#### The performance coded by independent judges

The same coding grid and procedure was used as the one used in the video test. Two independent trained judges (the same as for the video test coding) had to watch every role play's video recording and complete the coding grid. The judges' training and coding procedure was the same as for the video test. Each helper's sentence was coded into one of the seven exploration skills. Again, we divided the exploration helping skills by the total number of skills used during the role play to get the proportion of exploration helping skills; higher scores thus indicate higher use of helping skills compared to the total of their spoken interventions in the role play. As presented in Table 2, interrater reliabilities were satisfactory for the 7 helping skills. Students spontaneously used no summarizing and no situation clarification at pretest, such that an intra-class correlation coefficient could not be calculated.

**Table 2:** Intra-class correlation coefficients for helpers' helping skills used during the role play, as coded by independent judges.

	**Pretest**	**Posttest**
1. Minimal encouragement	0.91	0.96
2. Asking questions	0.85	0.92
3. Paraphrasing	0.84	0.80
4. Reflection of feeling	0.80	0.90
5. Concreteness	0.86	0.82
6. Summarizing	/	1.0
7. Situation clarification	/	0.86

#### The performance rated by helpers and their helpees

##### The performed helping skills

Helpers' helping skills performed in the role play self- and peer-reported were assessed with the Helping Skills Measure (HSM; Hill and Kellems, 2002). This questionnaire contains 13 items assessing the helper's use of helping skills during a helping session (or role-play) at every stage of the helping skills model: exploration, insight and action. Items are the same in the helper- and helpee-reported version. For example, the first item in the self-reported version is “*In this session, I asked questions to help the helpee explore what s/he was thinking or feeling*,” and in the helpee-reported version “*In this session, my helper asked questions to help me explore what I was thinking or feeling*.” Answers are given on five-point scales ranging from 1 (*strongly disagree*) to 5 (*strongly agree*). The HSM can provide specific scores for the three helping stages, as well as a total helping skills' score. Theses scores are obtained by summing the items, higher scores thus indicating higher levels of perceived helping skills in the role play. The HSM has been validated in Hill and Kellems (2002). In the present study, the HSM was translated into French by our team (using the back-translation method). As in Goates-Jones (2004), the exploration helping skills subscales had unsatisfactory internal consistency alpha's, leading us to use the total score. Internal consistency alphas for the total HSM at the pretest were 0.83 for helpers and 0.87 for helpees. At posttest, they were 0.78 for helpers and 0.78 for helpees.

##### The performed session quality

To assess the perceived quality of the role played session (helpful, valuable, satisfying, and effective), the Session Evaluation Scale (SES; Hill and Kellems, 2002) was completed both by the helper and the helpee as it exists in self- and peer-reported versions (e.g., “*I/my client thought that this session was helpful*”). Items are rated on a five-point scale ranging from 1 (*strongly disagree*) to 5 (*strongly agree*) and summed to get the SES total score, higher scores thus indicating higher levels of perceived session quality during the role play. We translated the five-item version of the SES into French, using the back-translation method. In the present study, internal consistency alphas at the pretest were 0.80 for helpers and 0.89 for helpees. At posttest, they were 0.82 for helpers and 0.92 for helpees.

##### The performed therapeutic relationship

The Relationship Scale (RS; Hill and Kellems, 2002) provides a self- and peer-reported assessment of the therapeutic relationship (trust, bond, collaboration, agreeability) between the helper and the helpee during a session (e.g., “*In this session, I did not feel a bond with my helper*” or “*In this session, my client did not feel a bond with me*”). It includes four items scored on a five-point scale ranging from 1 (*strongly disagree*) to 5 (*strongly agree*) that are summed to obtain a total RS score, higher scores thus indicating higher levels of perceived therapeutic relationship during the role play. We translated the RS into French (using the back-translation method) and asked the helpers and helpees to complete it just after the role play. The internal consistency alphas at pretest were 0.62 for helpers and 0.89 for helpees. At posttest, they were 0.76 for helpers and 0.92 for helpees.

##### The performed therapeutic attitudes

Helpers' empathy, warmth, and genuineness during the role play were assessed with the Empathy Scale's (ES; Burns et al., 1996) French version (Brison et al., 2015). It includes 10 items rated on a four-point scale ranging from 0 (*not at all*) to 3 (*a lot*) and evaluating the helper's therapeutic attitudes toward the helpee during the session. We used both the helper's (self-reported, e.g., “*My patient felt understood during today's session*”) and the helpee's versions (peer-reported, e.g., “*My therapist understood what I said during today's session*”) after the role plays. The total ES score is obtained by adding the first five items (formulated positively) and subtracting the last five items (formulated negatively). Scores can range from -15 (lowest empathy rating) to +15 (highest empathy rating). Internal consistency alphas at pretest were 0.66 for helpers and 0.84 for helpees. At posttest, they were 0.58^3^ for helpers and 0.83 for helpees.

### Data analyses

First, to examine whether helpers improved their helping skills after the training course as assessed by objective and subjective measures (= hypothesis 1), we conducted 2 (helping skills training vs. control group) × 2 (pretest vs. posttest) repeated measure analyses of variance (ANOVA), with condition as the between-subject variable and testing time as the within-subject variable. Effect sizes are reported as partial eta squareds ($\eta_{p}^{2}$) with a significance level set at 0.05. We expected interactions between condition and testing time indicating a bigger improvement in one condition than the other. As we expected this improvement to occur in the training group, and not in the control group, we analyzed if score differences between the pre- and the posttests were significant in both groups separately. Student's paired-samples *t*-tests were used. To control for multiple comparisons, the level of alpha (0.05) was corrected by the number of measures (5), leading to a significance level of 0.01. Effect sizes (Cohen's d) were calculated by dividing the mean differences between pre- and posttests by their respective standard deviations, with *d* = 0.2–0.49 representing a small, *d* = 0.5–0.79 a medium, and *d* ≥ 0.8 a large effect (Cohen, 1988). A series of initial independent *t*-tests confirmed that there was no significant difference between helpers from the training course and the control group on any of the pretest measures, except for therapeutic attitudes (ES). As this variable is only used for the comparison between helpers' and their helpees' assessments inside the conditions, results should not be biased by this difference between conditions at pretest.

Second, to check whether changes in potential self-assessment bias occurred as a result of training (= hypothesis 2), we looked at discrepancies between helpees' and helpers' perceptions over time. The four performed variables that were rated by both helpees and helpers, i.e., performed helping skills (HSM), performed therapeutic relationship (SES), performed session quality (RS), and performed therapeutic attitudes (ES), were subjected to repeated measure analyses of variance, with condition (training vs. control group) as a between-subject factor and testing time (pretest vs. posttest) and evaluator's perspective (helper vs. helpee's evaluation) as within-subject factors. Effect sizes are reported as partial eta squared ($\eta_{p}^{2}$) with a significance level set at 0.05. We expected significant three-way interactions for the four measures. To more precisely analyze helpers' biases before (= hypothesis 2.1) and after the training (= hypothesis 2.2), independent samples *t*-tests were systematically used to compare mean differences between helpers' and helpees' ratings, separately in the control and training groups. Finally, to ensure that a potential helper's bias at posttest could be attributed to the training effect, we verified with paired samples *t*-tests whether trained helpers improved. To control for multiple comparisons, the level of alpha (0.05) was corrected by the number of measures (4), leading to a significance level of 0.01.

## Results

### Are helpers more helping skilled after the training course? (= hypothesis 1)

As shown in Table 3, there was an improvement in all objective helping skills measures in the trained group. We observed significant interactions between condition and testing time for each level of acquisition of objective helping skills: helping skills knowledge, *F*_(1, 69)_ = 28.69, *p* < 0.001, $\eta_{p}^{2}$ = 0.29, helping skills used during the video test (competence level), *F*_(1, 69)_ = 13.66, *p* < 0.001, $\eta_{p}^{2}$ = 0.17, and helping skills used during the role-play (performance level), *F*_(1, 69)_ = 31.49, *p* < 0.001, $\eta_{p}^{2}$ = 0.31. Paired samples *t*-tests confirmed that trained helpers significantly improved their objectively assessed helping skills while untrained helpers did not, except for the competence level where untrained helpers also improved, *t*_(33)_ = −2.78, *p* < 0.01, *d* = 0.48, but less than trained helpers, *t*_(34)_ = −9.06, *p* < 0.001, *d* = 1.50. However, contrary to our expectations, we found no interaction between condition and testing time for helpers' and helpees' subjective ratings of helpers performed helping skills. Actually, there was no significant ANOVA effect for helpers' and helpees' ratings. In conclusion, our hypothesis 1 of a trained helpers helping skills' improvement is confirmed by the objective measures but not by helpers' or helpees' ratings.

**Table 3:** Mean (SD in parentheses) of the full factorial condition by time effects (repeated measure ANOVA) on helping skills measures.

	**Condition**	**Time**		**Repeated measure ANOVA effects**^a^					
		**Pretest**	**Posttest**	**Time**	**$\eta_{p}^{2}$**	**Condition**	**$\eta_{p}^{2}$**	**Condition by time**	**$\eta_{p}^{2}$**
**OBJECTIVE HELPING SKILLS**									
Knowledge	Training	6.52 (0.90)	8.10 (0.92)	35.81^***^	0.34	15.54^***^	0.18	28.69^***^	0.29
	Control	6.07 (1.28)	6.39 (1.09)						
Competence	Training	0.38 (0.17)	0.71 (0.16)	63.55^***^	0.49	25.84^***^	0.28	13.66^***^	0.17
	Control	0.32 (0.20)	0.44 (0.19)						
Performance	Training	0.38 (0.18)	0.68 (0.15)	50.26^***^	0.42	14.66^***^	0.17	31.49^***^	0.31
	Control	0.40 (0.17)	0.43 (0.17)						
**SUBJECTIVE HELPING SKILLS**									
Helper-reported	Training	40.84 (8.7)	40.28 (6.51)	0.35	0.01	3.86	0.05	0.56	0.01
	Control	43.61 (6.68)	43.86 (6.82)						
Helpee-reported	Training	37.6 (7.34)	42.28 (7.59)	2.88	0.04	0.81	0.01	1.7	0.02
	Control	38.20 (11.94)	38.89 (8.58)						

*** *p < 0.001*.

a *The F-value was calculated on the basis of df = (1, 67) to (1, 69) depending on the outcome*.

### Do helpers have a self-assessment bias? (= hypothesis 2)

**Figure 2:**
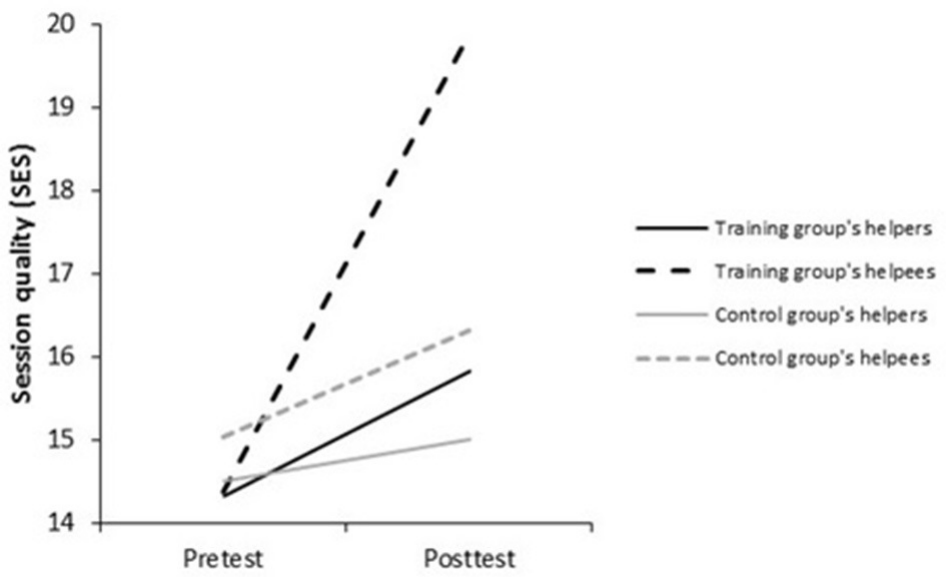
Session quality performed during the role play (SES), as reported by helpers and helpees at pre- and posttest.

**Figure 3:**
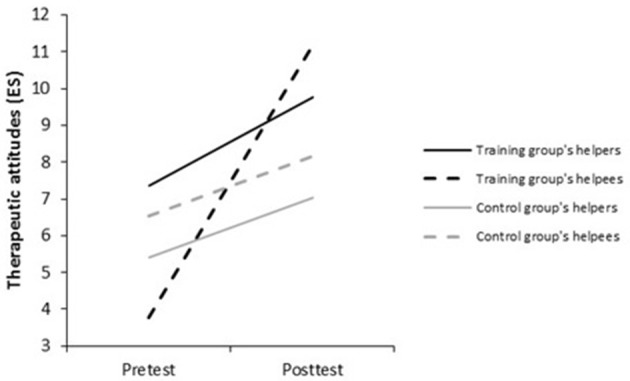
Helpers' therapeutic attitudes performed during the role play (ES), as reported by helpers and helpees at pre- and posttest.

**Figure 4:**
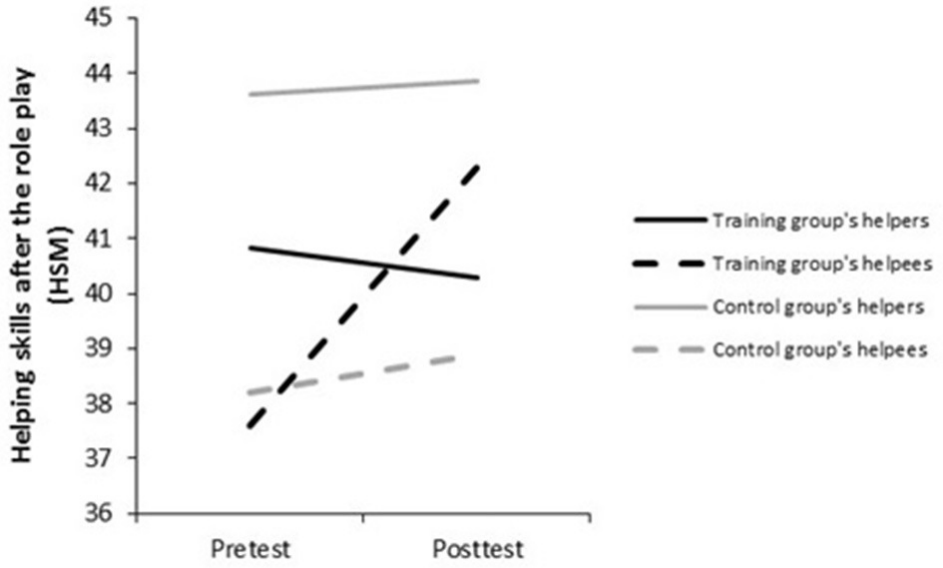
Helpers' performed helping skills during the role play (HSM), as reported by helpers and helpees at pre- and posttest.

**Figure 5:**
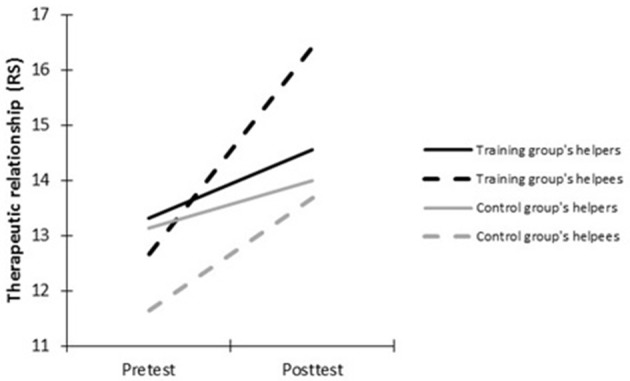
Therapeutic relationship performed during the role play (RS), as reported by helpers and helpees at pre- and posttest.

Three-way significant interactions (Condition × Testing Time × Evaluator) were found for two of the four variables: the performed session quality (SES), *F*_(1, 69)_ = 5.03, *p* < 0.03, $\eta_{p}^{2}$ = 0.07, and the performed therapeutic attitudes (ES), *F*_(1, 69)_ = 5.22, *p* < 0.02, $\eta_{p}^{2}$ = 0.07. Finally, no triple interaction was found neither for the performed helping skills rated by helpers and their helpees (HSM), *F*_(1, 69)_ = 2.71; *p* < 0.1; η^2^ = 0.04, nor for the performed therapeutic relationship (RS), *F*_(1, 69)_ = 1.12, *p* < 0.29, $\eta_{p}^{2}$ = 0.02. Results are depicted in Figures 2–**5**, respectively. The three-way interactions (Condition by Testing Time by Evaluator) indicated that training influenced the variables differently over time depending on evaluators' perspective. Means and repeated measure ANOVA effects are detailed in Table 3 for the performed helping skills and in Table 4 for the performed session quality, therapeutic attitudes and therapeutic relationship. Table 5 shows helpers' biases for these four dependent variables. Results will be detailed for each variable separately

**Table 4:** Mean (SD in parentheses) of the full factorial condition by time effects (repeated measure ANOVA) on performed session quality, therapeutic relationship and therapeutic attitudes.

**Variables**	**Condition**	**Time**		**Repeated measure ANOVA effects**^a^					
		**Pretest**	**Posttest**	**Time**	**$\eta_{p}^{2}$**	**Condition**	**$\eta_{p}^{2}$**	**Condition by Time**	**$\eta_{p}^{2}$**
**SESSION QUALITY (SES)**									
Helper-reported	Training	14.51 (3.71)	15.83 (3.22)	3.40	0.05	0.42	0.01	0.72	0.01
	Control	14.51 (3.35)	15.00 (3.18)						
Helpee-reported	Training	14.53 (4.36)	19.56 (4.00)	17.72^***^	0.20	3.05	0.04	6.38^*^	0.08
	Control	15.06 (5.03)	16.31 (4.71)						
**THERAPEUTIC RELATIONSHIP (RS)**									
Helper-reported	Training	13.47 (1.84)	14.56 (2.23)	12.56^**^	0.15	1.27	0.02	0.17	0.00
	Control	13.14 (2.11)	14.00 (1.83)						
Helpee-reported	Training	12.92 (3.48)	16.42 (2.99)	24.28^***^	0.26	12.22^**^	0.15	1.56	0.02
	Control	11.60 (3.46)	13.68 (3.69)						
**THERAPEUTIC ATTITUDES (ES)**									
Helper-reported	Training	7.53 (3.48)	9.75 (2.46)	21.50^***^	0.24	13.40^***^	0.16	0.77	0.01
	Control	5.51 (3.77)	7.03 (2.96)						
Helpee-reported	Training	4.22 (4.73)	11.17 (3.39)	24.42^***^	0.26	0.22	0.00	9.23^**^	0.12
	Control	6.51 (5.29)	8.17 (5.69)						

*SES, Session Evaluation Scale; RS, Relationship Scale; ES, Empathy Scale*.

* *p < 0.05*,

** *p < 0.01*,

*** *p < 0.001*.

a *The F-value was calculated on the basis of df = (1, 68) to (1, 69) depending on the outcome*.

**Table 5:** Summary of the comparisons between helpers' and their helpees' assessments at pre- and posttest in the control and in the training groups, for performed helping skills, session quality, therapeutic relationship and therapeutic attitudes.

**Variables**	**Condition**	**Time**	
		**Pretest**	**Posttest**
Helping skills (HSM)	Training	Helpers > Helpees	Helpers = Helpees
	Control	Helpers > Helpees	Helpers > Helpees
Session quality (SES)	Training	Helpers = Helpees	Helpers < Helpees
	Control	Helpers = Helpees	Helpers = Helpees
Therapeutic relationship (RS)	Training	Helpers = Helpees	Helpers < Helpees
	Control	Helpers = Helpees	Helpers = Helpees
Therapeutic attitudes (ES)	Training	Helpers > Helpees	Helpers = Helpees
	Control	Helpers = Helpees	Helpers = Helpees

*HSM, Helping Skills Measure; SES, Session Evaluation Scale; RS, Relationship Scale; ES, Empathy Scale*.

First, at pretest, there was no significant difference between helpers' and helpees' ratings neither in the training group, nor in the control group for the performed session quality (SES) (*ns*). Over time, in the training group, helpers reported no improvement in performed session quality (*ns*), while helpees perceived a large improvement in performed session quality, *t*_(35)_ = −5.28, *p* < 0.001, *d* = −0.88. In the control group, no improvement was perceived from both evaluator perspectives (*ns*). As a result, at posttest, helpers from the training group evaluated performed session quality as being worse than the helpees did, *t*_(34)_ = −5.33, *p* < 0.001, *d* = −1.2. In the control group, no significant difference was found at posttest in the evaluations of performed session quality according to evaluator perspective (*ns*) (see Figure 2, Tables 4, 5). In conclusion, contrary to our expectations, at pretest, there was no self-enhancement bias about performed session quality but rather an agreement between helpers and helpees (2.1). However, as expected, a self-diminishment bias occurred at posttest among helpers from the training group, while the agreement between helpers and helpees remained in the control group. Therefore, the second hypothesis can be confirmed (2.2): trained helpers display a self-diminishment bias concerning performed session quality.

Second, with regard to helpers' performed therapeutic attitudes (ES) at pretest, the control and training groups were not equivalent. In the training group, while helpers assessed their performed therapeutic attitudes as higher than their helpees did, *t*_(36)_ = 3.51, *p* < 0.001, *d* = 0.80, there was no difference between helpees and helpers' evaluations in the control group (*ns*). Over time, in the training group, helpers perceived an improvement in their performed therapeutic attitudes, *t*_(35)_ = −3.97, *p* < 0.001, *d* = −0.68, but less than the helpees did, *t*_(35)_ = −7.00, *p* < 0.001, *d* = −1.18. In the control group, no improvement was perceived from both evaluator perspectives (*ns*). At posttest, training group helpers' performed therapeutic attitudes were assessed equally by helpers and helpees (*ns*). In the control group, there remained no difference between helpers' and helpees' evaluations (*ns*) (see Figure 3, Tables 4, 5). To sum up, there was a self-enhancement bias for helpers in the training group at pretest concerning their therapeutic attitudes (hypothesis 2.1). Contrary to our expectations, this did not occur in the control group where helpers and helpees' assessments were equivalent. The second hypothesis can only be partly confirmed (hypothesis 2.2): trained helpers stopped to overestimate their performed therapeutic attitudes as a training effect.

Third, with regard to the performed helping skills rated by helpers and their helpees (HSM), even if the three-way interaction was not significant, the observed trends in *post-hoc* tests were consistent with those on the other dependent variables (see Figure 4, Tables 3, 5). Helpers provided a better evaluation of their own performed helping skills at pretest than the helpees did both in the training group, *t*_(36)_ = 3.47, *p* < 0.001, *d* = 0.52, and the control group, *t*_(35)_ = 2.69, *p* < 0.01, *d* = 0.60. At posttest, this trend remained in the control group where helpers continued to rate themselves as better than the helpees did, *t*_(34)_ = 2.72, *p* < 0.01, *d* = 0.65. In the training group, helpees' ratings of helpers' performed helping skills became equal to helpers' ratings (*ns*). Over time, helpers from the training group perceived no improvement of their performed helping skills (*ns*), whereas helpees did, *t*_(35)_ = −3.24, *p* < 0.01, *d* = −0.48. In the control group, both helpers and helpees reported no improvement (*ns*) in performed helping skills during the role play. To sum up, as expected, there was a helper self-enhancement bias about performed helping skills during the role play at pretest (hypothesis 2.1). This helpers' self-enhancement bias was maintained at posttest for helpers who were not trained and stopped for those who were trained, their self-ratings becoming equal to their helpees' ratings. The second hypothesis can tentatively be partly confirmed (hypothesis 2.2): trained helpers stopped to overestimate their performed helping skills as a training effect.

Finally, with regard to performed therapeutic relationship (RS), even if the three-way interaction was not significant, the observed trends in *post-hoc* tests were consistent with those on the other dependent variables. At pretest, there was no significant difference between helpers' and helpees' ratings neither in the training group, nor in the control group for the performed therapeutic relationship (*ns*). Over time, in the training group, helpers reported no improvement in performed therapeutic relationship (*ns*), while helpees did, *t*_(35)_ = −4.63, *p* < 0.001, *d* = −0.77. In the control group, no improvement was perceived from both evaluator perspectives (*ns*). At posttest, helpees from the training group assessed the performed therapeutic relationship as being better than the helpers did, *t*_(35)_ = −3.74, *p* < 0.001, *d* = −0.72. In the control group, there remained no difference between helpees' and helpers' assessments at posttest (*ns*) (see Figure 5, Tables 4, 5). Thus, to sum up, the three-way interaction was not significant for the performed therapeutic relationship. However, results were consistent with previous results. In conclusion, contrary to our expectations, there was no self-enhancement bias about performed therapeutic relationship at pretest but an agreement between helpers and helpees (hypothesis 2.1). At posttest, a helpers' self-diminishment bias was present in the training group while assessments remained similar between helpers and helpees in the control group. In conclusion, the second hypothesis can tentatively be partly confirmed (hypothesis 2.2): trained helpers displayed a self-diminishment bias concerning the performed therapeutic relationship.

## Discussion

The aim of this study was to examine the validity and effectiveness of a helping skills training program both at improving novice helpers' helping skills and at reducing their self-enhancement bias. Firstly, we wanted to test whether helpers improved their helping skills after training, as assessed by different objective and subjective helping skills measures. Our hypothesis (1) of an improvement that would be reflected on all the helping skills measures was confirmed by objective measures but not by helpers and helpees ratings. Secondly, we wanted to investigate helpers' potential self-assessment bias concerning their helping skills performance as well as their correlates (i.e., session quality, therapeutic relationship, and therapeutic attitudes). We hypothesized that, at pretest, helpers would systematically make assessments higher than their helpees did (self-enhancement bias) concerning their performed helping skills and correlates (2.1). As helpers' assessments at pretest were most of the time equal to helpees' assessments, this hypothesis was not confirmed. At posttest, we expected a trained helpers' self-diminishment bias (2.2), which was partly confirmed by our results. Trained helpers assessed the performed therapeutic relationship and performed session quality as lower than their helpees did at posttest (self-diminishment bias). This helpers' underassessment was not significant for performed helping skills and performed therapeutic attitudes, leading to the conclusion of helpers and helpees provide equal ratings for these variables.

Three out of the five helping skills measures reflected an improvement after the 12-h training course (1). Students performed better after training at the three first levels of Miller's pyramid (Miller, 1990): helping skills knowledge (knowledge), helping skills used during the video test (competence), and helping skills used during the role play (performance). For performed helping skills during the role play, an improvement was also significantly reported by the helpees, as reflected by paired samples *t*-tests, but not by repeated measure analyses of variance (ANOVA). Finally, helpers reported the same helping skills' mean scores at pre- and posttest. It suggests that training enabled helpers to become more skilled but, consistently with our second hypothesis (2.2), it also led them to underestimate their skills. Therefore, it indicates that testing the effectiveness of a helping skills training program requires the use of other measures than self-ratings.

The second hypothesis concerned the self-assessment bias of novice helpers before training. At pretest, we expected that helpers would systematically better evaluate their helping skills and correlates than helpees would (self-enhancement bias) (2.1). This was the case for the performed helping skills rated by helpers and their helpees in both conditions. The comparison of helpers' self-ratings to those from their helpees suggest that untrained novice helpers overestimate their performed helping skills. This is in line with Lepkowski et al. (2009), Little et al. (2005), and Urbani et al. (2002) showing that therapists overestimate their helping skills performance in comparison to judges' ratings before the training. However, for the helping skills' correlates, results were less clear cut. There was a helpers' self-enhancement bias for the performed therapeutic attitudes measure in the training group, but not in the control group. Contrary to expectations, ratings were equivalent between helpers and helpees for the remaining variables and conditions: performed session quality and performed therapeutic relationship in both conditions, as well as performed therapeutic attitudes in the control group. Even if the self-enhancement bias was not consistently found at pretest, it should be noted that helpers never assessed their performance as being lower than their helpees did on any of the pretested variables. This indicates that novice helpers clearly do not underestimate their skills at baseline and may lack of professional self-doubt (Nissen-Lie et al., 2015).

With regard to posttest results, there was a trained helper's self-diminishment bias for performed session quality and performed therapeutic relationship. For performed helping skills and performed therapeutic attitudes, helper's and helpee's rating were not significantly different. Therefore, contrary to Lepkowski et al. (2009), Little et al. (2005), and Urbani et al. (2002), there was no significant self-diminishment bias for performed helping skills after the training. However, these authors used judges' ratings as a reference while we used helpees' ratings. Although posttest's self-diminishment bias was only confirmed for two out of the four measures, the position of helpers' ratings, as compared to helpees' ratings, decreased systematically at posttest. Trained helpers went either from a pretest overestimation to a posttest equivalence (performed helping skills and performed therapeutic attitudes), or from a pretest equivalence to a posttest underestimation (performed session quality and performed therapeutic relationship). Regarding untrained helpers, helpers' ratings position, as compared to helpees' ratings, remained exactly the same between pre- and posttest for the four variables: helpers continued to demonstrate either an overestimation of their performance (performed helping skills) or an equivalence with helpees' ratings (performed session quality, therapeutic relationship and therapeutic attitudes). As helpees reported an improvement of the four measures for trained helpers but not for the untrained ones, this might be explained by the training making helpers more skilled and therefore less confident. Thus, our results illustrate to some extent the skilled-unaware pattern (Burson et al., 2006).

Our results have two implications. First, assessing the effectiveness of helping skills training only using self-reported measures is insufficient. It probably results in underestimating its effects. To avoid this bias, a helping skills' multilevel evaluation is needed (Hill et al., 2008). It should especially include objective measures since they better reflected the improvement in helping skills than subjective ones. Second, helpers seemed somewhat unsettled just after training. Being confronted with the complexity of helping and with potentially negative feedback from their helpees, peers, and supervisors may have shattered their own perceptions and altered their favorable views of their skills (Kruger and Dunning, 1999). Whereas therapists' self-doubt is positively associated with alliance and outcome (Nissen-Lie et al., 2015), inexperienced helpers that are too unconfident after the training could focus more on themselves than on their helpees during helping sessions (Stoltenberg and McNeill, 2010). Therefore, efforts should be made during training and throughout follow-up, to reassure helpers about their competences, particularly if they have to conduct helping sessions with real clients shortly after training. However, it seems that helpers can get some confidence back over time, since Lepkowski et al. (2009) have shown that 29 months after training, helpers became accurate in their self-assessments.

Albeit interesting, the foregoing results need to be interpreted in light of the following limitations. First, as the three-way interaction aimed at analyzing helpers' and helpees' assessments' discrepancy over time was not significant for performed helping skills and performed therapeutic relationship, we checked whether it could be explained by a lack of power in our study. *Post-hoc* power analyses using GPower (Erdfelder et al., 1996) were conducted for ANOVA repeated measures (within-between interactions), with α = 05 (two-tailed), power (1 - β) set at 0.80, two groups and two measurements. For performed helping skills, the three-way interaction achieved effect size f(U) = 0.20 (based on $\eta_{p}^{2}$ = 0.04), would have required 204 participants to reach significance at the 0.05 level. For performed therapeutic relationship, the three-way interaction achieved effect size f(U) = 0.13 (based on $\eta_{p}^{2}$ = 0.02), would have required 488 participants to reach significance at the 0.05 level. As such samples sizes are pretty unreasonable in the helping skills training research's field, it seems impossible to reliably prove such three-way interactions. Thus, failure of finding an effect might be due to this study's sample size. Finally, for the detection of small effect sizes ($\eta_{p}^{2}$ = 0.02), a sample of 122 participants would have been required. Second, helpers in the control group unexpectedly significantly improved on three outcomes out of 11: helping skills in the video test, helpee-reported therapeutic relationship and helper-reported therapeutic attitudes in the role play. This suggests a repeated test learning effect that was also found using repeated video tests (Kuntze et al., 2007) and role plays (Brison et al., 2015). Moreover, helpers from the training and control groups were not equivalent at baseline on their therapeutic attitudes. Third, the absence of a posttest in the longer term prevents from checking whether the results remained over time and how long for. Fourth, the lack of evaluation of Miller's pyramid's fourth level (action stage) (Miller, 1990) does not enable to know whether helpers would be able to use their helping skills in a real helping session with a real helpee. Fifth, the Cronbach's alphas for helpers' ratings of performed therapeutic attitudes and performed therapeutic relationship were low at pretest (0.66, 0.62) and posttest (0.58, 0.76), respectively. Since the impact of these poor alphas on the results is unclear, the conclusions that can be drawn concerning these variables are limited. Sixth, as it is often the case in the helping skills' training field (Hill and Lent, 2006), students were not randomly assigned to the training and control conditions.

## Concluding comment

Twelve hours of training enabled novice helpers to improve their objective helping skills. Furthermore, results showed an improvement in self-assessed session quality and tentatively suggest an improvement in perceived helping skills, therapeutic attitudes and therapeutic relationships. It is likely that, by becoming more skilled, they became also more self-critical about their level of acquired helping skills and performance.

## Author contributions

MJ conceived and conducted the research, analyzed and interpreted the data for the article and wrote the article, EZ gave a major contribution to the analysis and interpretation of data for the article, and fully revised the article critically multiple times, CB contributed to the research's conception and conduction and to data analysis, LB contributed to the research's conception and participated to meetings about data analysis and interpretation, NV contributed to the training's creation and participated to meetings about data analysis and interpretation, MM contributed critically to the analysis and interpretation of data for the article, and revised the article.

### Conflict of interest statement

The authors declare that the research was conducted in the absence of any commercial or financial relationships that could be construed as a potential conflict of interest.

## Footnotes

^1^They were trained to give the helping skills course by the first author. They were provided with theoretical information about the content of the course as well as course exercises, slides, and paper and video supports. They then had to practice training in front of their supervisor. This supervisor was present during the first session of both subgroups and acted as a co-trainer. Trainers received feedback to improve their training sessions with students.

^2^They received two hours' theoretical training about helping skills and then had to code a copy orally in front of their supervisor. Afterwards, they both had to code the answers of five participants. Their supervisor looked at their coding sheets, calculated the intra-class correlation coefficients for each category and gave them a feedback to improve their coding.

^3^Since the same version was used at both times and both perspectives (only changing “I” in “my helper”) and as this poor alpha occurred only in the self-reported version at post-test, it cannot be explained by a problem of the scale. Alpha did not improve when removing any of the items. This will be addressed in the discussion.
